# Effects of Chemical Post-treatments on Structural and Physicochemical Properties of Silk Fibroin Films Obtained From Silk Fibrous Waste

**DOI:** 10.3389/fbioe.2020.523949

**Published:** 2020-12-02

**Authors:** Melissa Puerta, Maria S. Peresin, Adriana Restrepo-Osorio

**Affiliations:** ^1^Grupo de Investigación Sobre Nuevos Materiales, Universidad Pontificia Bolivariana, Medellín, Colombia; ^2^Forest Products Development Center, School of Forestry and Wildlife Sciences, Auburn University, Auburn, AL, United States; ^3^Facultad de Ingeniería Textil, Escuela de Ingenierías, Universidad Pontificia Bolivariana, Medellín, Colombia

**Keywords:** silk fibroin, silk fibrous waste, alcohol post-treatment, secondary structure, water stability

## Abstract

Silk fibroin (SF) is a protein polymer claimed to have outstanding potential for medical applications. However, because of the manufacturing process, materials from regenerated SF exhibit a higher percentage of amorphous structures. The amorphous structures cause the material to be water soluble and can significantly limit its applications in wet biological environments. In order to increase the amount of crystalline structures and decrease the water solubility of SF materials, post-treatment with alcohols is usually employed. SF can be obtained from silk fibrous wastes (SFW), usually discarded in silk textile processes. This represents an opportunity to produce materials with high added value from low-cost natural sources. In this study, SF was obtained from SFW, and films were made thereof followed by a post-treatment by immersion or in a saturated atmosphere of methanol (MeOH) or ethanol (EtOH), using different exposure times. The resulting films were analyzed according to crystallinity, the percentage of crystalline and amorphous structures, and thermal stability. Also, water absorption and weight loss in aqueous media were determined. The results showed a significant increase in crystalline structures in all treated samples, varying according to the type and time of exposure to post-treatment conducted. The highest increase was shown in the case of the post-treatment by immersion in MeOH for 1 h, with a 23% increase over the untreated sample. This increase in crystallinity was reflected in an increase in the degradation temperature and a degradation rate of 5.3% on day 7. The possibility of tuning the degree of crystallinity, as well as thermal stability and aqueous integrity of thin films of SFW, can be applied to adjust these materials to the requirements of specific biomedical applications.

## Introduction

Silk fibroin (SF) is a protein that can be extracted from the silk produced by the *Bombyx mori* silkworms. In recent years, SF has been studied for medical applications such as cutaneous wound healing (Bhardwaj et al., 2015; Panico et al., 2018), bone tissue regeneration (Ko et al., 2016), and vascular implants (Bonani et al., 2011; Wu et al., 2018), among others. This has been made possible thanks to its biocompatibility (Adali and Uncu, 2016), biodegradability, adequate mechanical performance (Fukayama et al., 2015), ability to promote cell growth and interaction (Unger et al., 2004), and its hemocompatibility and cytocompatibility (Fukayama et al., 2015; Adali and Uncu, 2016). SF is usually extracted from cocoons of high-quality silkworms (Rockwood et al., 2011; Tao et al., 2012; Huang et al., 2017) but there are other sources such as silk fibrous wastes (SFW). In Colombia, this waste is widely available as raw material and is provided by a few small-scale sericulture production units. Many of these production units are interested in finding alternative uses to increase the value of this waste stream. Such is the case of the Corporation for the Development of Sericulture of Cauca (CORSEDA), which produces about 20 tons/year of cocoons. It is estimated that the amount of fibrous by-products of silk can reach up to 30% by weight of the cocoons produced, in which SF is the predominant protein (Babu, 2013; Jaramillo-Quiceno and Restrepo-Osorio, 2019). The development of new value-added products from waste streams, such as the manufacture of materials from SF for medical applications, may represent an alternative solution for increasing the sustainability of the sericulture chain.

On the other hand, one of the most relevant factors for materials used for biomedical purposes is their controlled biodegradability, as this defines the possible functions and durability according to the specific application of a biomaterial (Kim et al., 2012). Due to processing methods, materials manufactured from regenerated SF may have a higher percentage of amorphous structures including random coil, α helix, side chains, turns, and bends. These structures are less orderly and have weak bonds, making the material water soluble and giving the SF reduced mechanical properties (Kim et al., 2012; Babu, 2013). Therefore, post-treatments are required to enrich and control the quantity of crystalline structures of the SF, known as β sheets and β-turns, which allows for tuning of properties including thermal stability and integrity in aqueous media. Some SF post-treatments include vapor or immersion of solvents such as EtOH and MeOH, separated or mixed, to modify its crystalline structure and decrease its water solubility (Jeong et al., 2006; Zhang et al., 2009; Kim et al., 2012; Terada et al., 2016).

There are few reports of SFW as raw materials for biomaterials fabrication. Also, no further literature reports the post-treatment methods presented here, immersion and atmospheric saturation with ethanol (EtOH) and methanol (MeOH) as solvents, and their comparative effect on SF properties. In this work, we investigate the effect of post-treatment of SFW using EtOH and MeOH. The followed methodology was immersion and exposure of SFW to atmosphere saturated in EtOH and MeOH for 15 min and 1 h, independently for each treatment. Morphological, thermal, and chemical properties of the materials, in addition to crystallinity, were investigated by X-ray diffraction (XRD), Fourier transform infrared spectroscopy with attenuated total reflectance (FTIR-ATR), scanning electron microscopy (SEM), differential scanning calorimetry (DSC), weight loss, and water absorption.

## Materials and Methods

### Silk Fibroin Extraction

**FIGURE 1:**
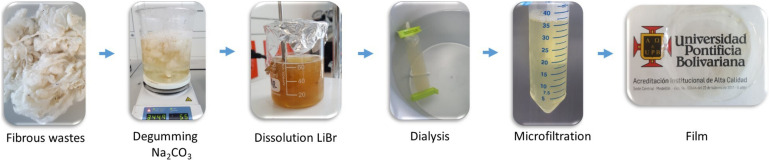
SFW extraction porcess.

Silk fibroin was extracted from fibrous wastes, provided by CORSEDA, following previously published procedures (Rockwood et al., 2011; Jaramillo-Quiceno et al., 2017; Jaramillo-Quiceno and Restrepo-Osorio, 2019). Briefly, the fibrous waste was degummed twice in a 0.5% w/w aqueous solution of Na_2_CO_3_ (MERK, Germany) at boiling point for 30 min. The obtained SFW was dried at 60°C for 24 h and subsequently dissolved using LiBr (Sigma-Aldrich, St. Louis, MO, United States, > 99% purity), 9.3 M at 60°C. The solution was dialyzed and finally microfiltered obtained an SFW aqueous solution at 5.6% w/w. Subsequently, films were prepared by solvent casting and dried at 35°C until constant weight was reached. The obtained films had thicknesses 115 ± 2 μm, determined with a micrometer device. The isolation process is illustrated in Figure 1.

### Post-treatments of Silk Fibroin Films

Post-treatment of the SFW films was performed using EtOH (SIGMA ALDRICH > 99.8% purity) and MeOH (EMSURE > 99.9% purity), by (i) immersing the films in or (ii) exposing them to a saturated atmosphere with vapors of each of the solvents. The samples will be referred to as the following: films immersed in MeOH (IMeOH), exposed to MeOH vapor (VMeOH), immersed in EtOH (IEtOH) and exposed to EtOH vapor (VEtOH). To determine the effect of post-treatment time, the properties were evaluated after 15 min and 1 h of immersion/vapor exposure. These times were chosen according to previous reports (Mandal et al., 2009; Bie et al., 2015; Srivastava et al., 2015; Zhou et al., 2016; Bagrov et al., 2017).

### Crystallinity

To determine the crystallinity of the samples with and without post-treatments, an XPert PANalytical Empyrean Series II diffractometer was used with a Cu source. XRD patterns were obtained in a range (2θ) between 5 and 50°, with a step of 0.026° and time per step of 50 s.

### Chemical Structure

The effect of post-treatments on the chemical structure of SFW films with and without post-treatments was evaluated by means of the FTIR technique (Nicolet 6700 Series). 64 scans and a resolution of 4 cm^–1^ were recorded in a range of the absorption spectrum between 4,000 and 400 cm^–1^. OMNIC software was used to deconvolute the spectra in the region of amide I (1,700–1,600 cm^–1^) as detailed in previous work (Jaramillo-Quiceno and Restrepo-Osorio, 2019). This spectral region was selected as it is the most sensitive to changes in the secondary structures of the protein which originate from the C = O stretch vibration of the amide groups together with the phase flexion of the N-H bond and C-N bond stretch (Yang et al., 2015).

### Thermal Behavior

To measure the thermal behavior of the films with and without post-treatments, DSC technique was used in a Q2000 TA Instruments unit. The samples were subjected to a temperature scan from 30 to 320°C and a heating rate of 10°C/min. In order to remove the water content in the samples and improve the baseline of thermogram, an isotherm at 120°C was performed prior to measurement in all cases.

### Absorption Water and Weight Loss in Aqueous Medium

The integrity of films in an aqueous medium was determined by absorption water and weight loss of the SF films with and without post-treatments. The assay was carried out through deposition of dry pieces, with a known weight and with an area approximately 1 cm^2^, in distilled water at 37°C. After 1 and 7 days, the samples were removed from the water, cleared of excess water, and weighed. The weight loss was determined by percentage of solubilized mass, according to Eq. 1.

(1) $$\%\text{WL}=\left({\text{W}}_{0}-{\text{W}}_{f}\right)/{\text{W}}_{0}$$

where, W_0_: initial weight, W_*f*_: final weight after water medium exposure of dried samples.

### Morphology

The effect of post-treatments and exposure to aqueous medium on morphology of the surface, and the cross-section of the films was observed with an SEM, JEOL JSM–6490 LV. Samples were cryofractured and sputtered with gold prior to the imaging. The acceleration voltage was 15 kV and approaches were made to ×2,000, ×5,000 for surface, and ×1,500 for cross-section images.

### Statistical Analysis

The effect of post-treatments on films crystallinity (FTIR-ATR), absorbed water and weight loss in water media test was assessed by applying ANOVA one way, with *n* = *3* for each assessed condition.

## Results and Discussion

### Crystallinity

**FIGURE 2:**
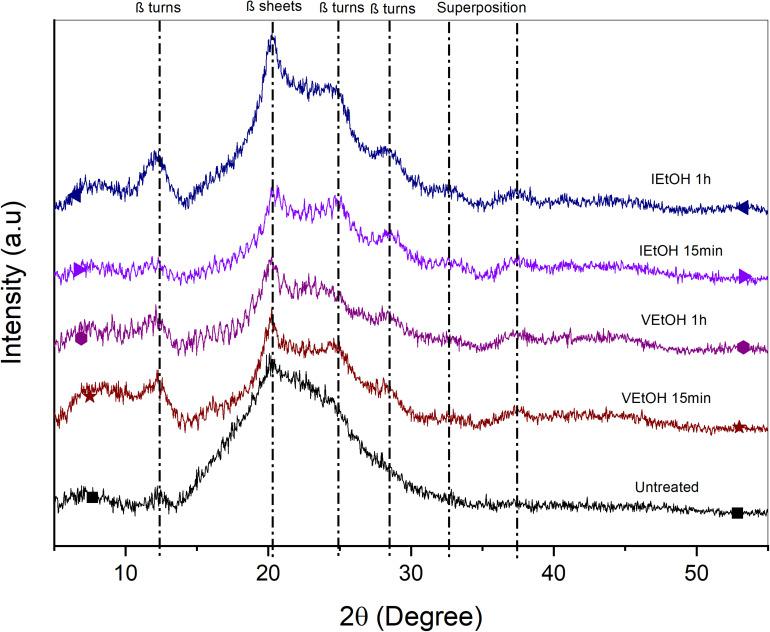
XRD untreated and treated films with EtOH.

**FIGURE 3:**
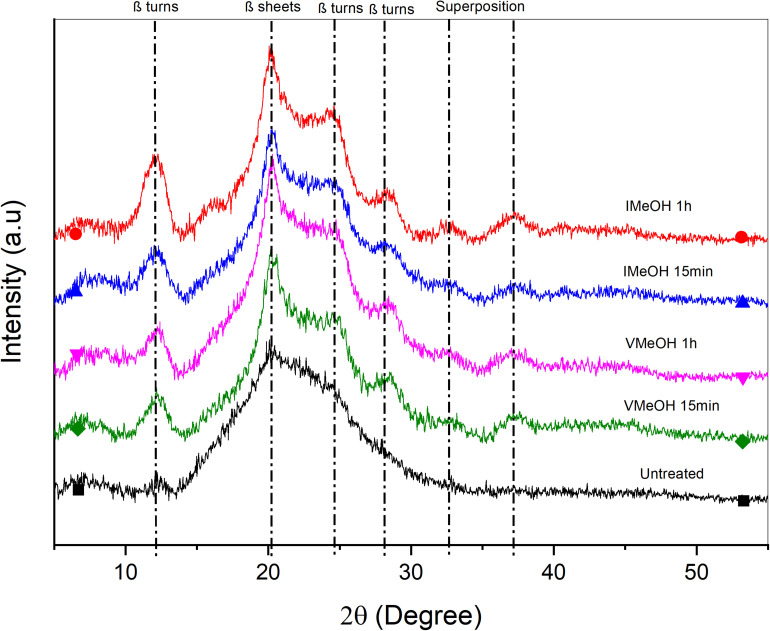
XRD untreated and treated films with MeOH.

X-ray diffraction was utilized to determine changes in crystallinity ratio attributed to post-treatment. Figures 2, 3 show the diffractograms of the films treated with EtOH and MeOH, respectively. Untreated films present a broad peak and low intensity at 2θ = 19.9° representative of random coil structures consistent with that reported in the literature (Nogueira et al., 2010). On the other hand, samples treated with MeOH and EtOH exhibit a defined peak at 2θ = 20.1° associated with β sheets (silk II) (Yan et al., 2012) and attenuated peaks at 2θ = 12.2°, 24.7°, and 28.6°corresponding to β turns (silk I) (Ha et al., 2005; Wongpanit et al., 2007; Yan et al., 2012; Zhou et al., 2016). In addition, there are two other peaks at 2θ = 32° and 37°, which can be attributed to the superposition of other specific peaks of the SF around 2θ = 24.71° (silk I), 18.40° (silk II), 20.14° (silk I), 21.54° (silk I), or 27.77° (silk I) (Jin and Kaplan, 2003; Stefan et al., 2019). The peaks in the samples treated with MeOH and EtOH have higher intensities compared to those observed in untreated samples, with the intensities in films treated with MeOH being relatively more pronounced. This suggests a higher degree of crystallinity in MeOH samples. The same behavior was presented in the work of Nogueira et al. (2010), where they compared the intensities of the untreated samples and those treated with EtOH.

**FIGURE 4:**
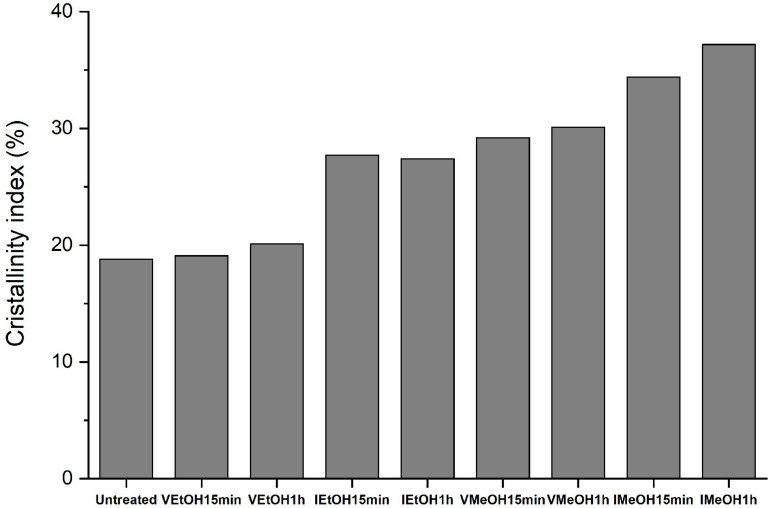
XRD crystallinity index of untreated and treated films.

The crystallinity index of the SFW films was calculated using the ratio of the area of the crystalline regions to the total diffraction area (Amiraliyan et al., 2010; Jaramillo-Quiceno and Restrepo-Osorio, 2019) and the results are present in Figure 4. According to the literature, the enrichment of crystalline structures was achieved through post-treatments, resulting in a greater effect on those samples treated with MeOH. This is attributable to the changes from amorphous structures such as random coil to β sheet reported for SF films treated by immersion in EtOH (Kaewprasit et al., 2018), MeOH (Çetin Altındal et al., 2019), and saturated vapor from both solvents (Jeong et al., 2006).

### Chemical Structure

Structural changes, attributable to different post-treatments, were studied by FTIR spectra analysis in the region within 1,700–1,200 cm^–1^ (Supplementary Figures 1, 2). This region is assigned to absorption of the peptide backbones of the Amide I (1,700–1,600 cm^–1^), the Amide II (1,600–1,500 cm^–1^), and the Amide III (1,350–1,200 cm^–1^). All treated samples showed a displacement of the evaluated bands (Table 1), indicating a change in the secondary structures from random coils to β sheets.

**TABLE 1:** Location of the peaks associated with the secondary structures in the SFW samples and the different treatments.

	**Random coil**	**β Turn**	**β Sheet**
Untreated	1,652, 1,538, and 1,240		
VEtOH 15 min	1,640, 1,525, and 1,236		
VEtOH 1 h	1,240	1,646 and 1,537	
IEtOH 15 min	1,642, 1,529, and 1230		1,618 and 1,512
IEtOH 1 h	1,242		1,622 and 1,515
VMeOH 15 min	1,642, 1,529, and 1,236		
VMeOH 1 h	1,510 and 1,231	1,618	
IMeOH 15 min	1,236		1,650 and 1,520
IMeOH 1 h	1,231		1,622 and 1,515

**FIGURE 5:**
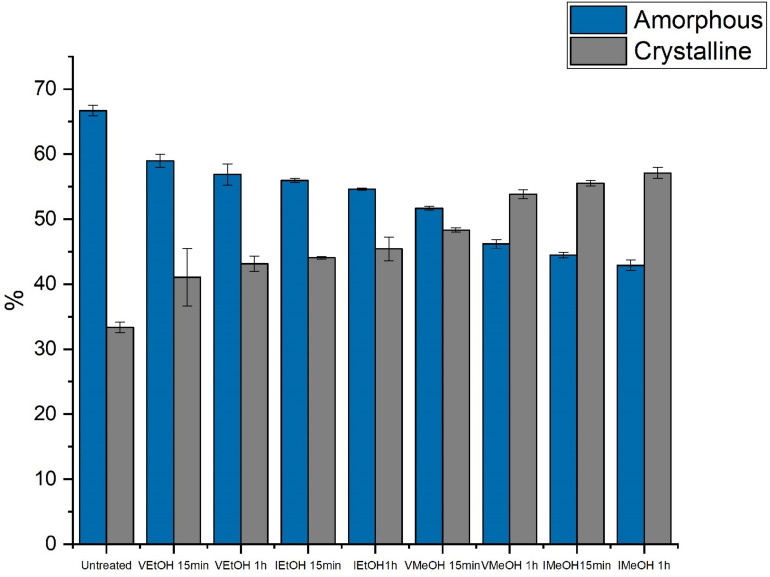
FTIR amorphous and crystalline structures of treated and untreated SFW films.

Additionally, the analysis of Amide I was performed using deconvolution of the peak to determine the proportions in the percentages of the secondary structures of the SFW films without treatment and when treated with EtOH and MeOH (Tables 2, 3). Figure 5 presents the total percentages of amorphous and crystalline structures in samples with and without post-treatments. The results obtained from the treated films indicate that all the post-treatments lead to an increase in crystallinity with respect to the untreated films. In all cases, p-values are <0.05, indicating statistically significant differences. The results obtained by FTIR confirm those found by means of XRD analysis. Specifically, a greater increase in crystallinity was observed due to post-treatment by immersion compared to with vapor saturated atmosphere. This can be attributed to the greater surface-to-surface interaction of film to solvent. Similarly, a higher percentage of crystalline structures was observed in samples treated by immersion in MeOH. This can be attributed to the greater polarity of MeOH in comparison to EtOH; as the formation of β sheets is favored by the contact of SFW with the solvent of greatest polarity (MeOH) (Jeong et al., 2006). The hydrogen bonds between the water molecules and SFW are rearranged into intermolecular hydrogen bonds within the SFW chains, due to the interaction between water and the organic solvent. EtOH has a lower affinity with water molecules than MeOH, so there is less formation of β sheets (Yazawa et al., 2018). Regarding the time of the post-treatments, it was found that 1 h caused a greater enrichment of crystalline structures. It is suggested that this is caused by a prolonged interaction between the solvents and the SF.

**TABLE 2:** Secondary structures percentages of SFW treated with EtOH and untreated films.

	**Untreated**	**VEtOH 15 min**	**VEtOH 1 h**	**IEtOH 15 min**	**IEtOH 1 h**
Side chains	14.3 ± 0.1	12.5 ± 1.8	12.8 ± 1.2	13.4 ± 0.2	14.8 ± 2.3
β Sheet	24.8 ± 0.8	29.0 ± 0.1	29.6 ± 0.4	33.7 ± 0.9	32.2 ± 3.1
Random coil	21.1 ± 0.4	17.8 ± 5.5	14.7 ± 2.1	11.1 ± 0.3	9.8 ± 2.3
α Helix	6.7 ± 0.0	7.5 ± 2.6	6.4 ± 1.9	8.9 ± 0.6	6.6 ± 2.4
β Turn	8.4 ± 0.0	11.9 ± 0.8	13.5 ± 1.2	10.3 ± 0.8	13.1 ± 3.5
Turns and bends	24.4 ± 0.1	21.0 ± 4.4	22.8 ± 1.5	22.5 ± 1.1	23.1 ± 1.9

**TABLE 3:** Secondary structures percentages of SFW treated with MeOH and untreated films.

	**Untreated**	**VMeOH 15 min**	**VMeOH 1 h**	**IMeOH 15 min**	**IMeOH 1 h**
Side chains	14.3 ± 0.1	7.3 ± 0.8	6.5 ± 0.2	6.9 ± 0.7	9.1 ± 0.8
β Sheet	24.8 ± 0.8	33.1 ± 0.3	35.7 ± 0.1	39.6 ± 0.9	42.4 ± 0.2
Random coil	21.1 ± 0.4	11.1 ± 0.9	11.0 ± 0.3	13.3 ± 0.5	9.1 ± 1.5
α Helix	6.7 ± 0.0	10.4 ± 1.4	10.0 ± 0.9	10.3 ± 0.1	6.9 ± 0.7
β Turn	8.4 ± 0.0	15.2 ± 0.5	18.0 ± 0.7	15.9 ± 0.8	14.6 ± 1.0
Turns and bends	24.4 ± 0.1	22.6 ± 1.1	18.5 ± 0.5	13.8 ± 0.2	17.6 ± 1.3

These findings are comparable with those reported by Chankow (2016) and Callone et al. (2016) who found increases in β sheets between 10 and 12% in SFW films treated with EtOH and MeOH. This behavior is attributed to the transformation of random coil into β sheets with post-treatments applied (Yali et al., 2014; Maghdouri-White et al., 2016). This is evidenced in the reduction of random coils in the treated samples compared with those untreated.

### Thermal Behavior

To examine the thermal properties, the DSC thermograms for the samples with and without treatment using EtOH or MeOH, are presented in Supplementary Figures 3, 4, respectively. The temperature values corresponding to the glass transition temperature (T_*g*_), the crystallization temperature, and the degradation temperature of the samples studied are included in Table 4. The exothermic recrystallization peak can be evidenced only in the samples without post-treatment and in VEtOH 15 min. In all the samples, the glass transition and endothermic degradation peaks occurred. In the treated samples there are two endothermic degradation peaks. The first peak is attributed to the degradation of amorphous structures while the second peak is attributed to the crystalline structures present in the material. In a sample without post-treatment, these two events are not completely differentiated.

**TABLE 4:** Summary of DSC data obtained from films with different post-treatments.

	**T_*g*_ (°C)**	**T_crystallization_ (°C)**	**T_degradation 1_ (°C)**	**T_degradation 2_ (°C)**
Untreated	144.30	229.59	255.80	272.98
VEtOH 15 min	151.20	231.80	258.13	273.47
VEtOH 1 h	151.61	–	256.65	273.05
IEtOH 15 min	152.75	–	258.00	275.32
IEtOH 1 h	153.11	–	258.11	273.12
VMeOH 15 min	153.90	–	256.72	274.37
VMeOH 1 h	160.73	–	259.47	274.29
IMeOH 15 min	164.45	–	257.01	274.82
IMeOH 1 h	165.92	–	260.10	276.73

The T_*g*_ occurs at a higher temperature in the treated samples compared to the samples without post-treatment. The highest one corresponding to IMeOH (165.92°C) compared to the untreated SFW (144.30°C), which in turn are the samples with highest and lowest crystallinity, respectively (McGill et al., 2018). This can be explained because, in the samples showing higher crystallinity, the protein chains are in a more compact state and need more energy for the molecular movement of the glass transition to occur (Park et al., 2011). The T_*g*_ values obtained in this study are relatively lower than those reported in the literature (Lu et al., 2010) which evaluates SF films obtained from silk cocoons and treated with immersion in MeOH. This can be due to the different raw material used in this work as compared to high quality cocoons used in the literature. SFW presents a secondary structure different from that obtained from silk cocoons, on account of the textile transformation processes to which it is subjected (Jaramillo-Quiceno et al., 2017).

As mentioned before, untreated films have a recrystallization peak and the film treated with VEtOH for 15 min showed a lower recrystallization peak than the untreated films, both near 230°C. This indicates that there are amorphous secondary structures that can crystallize due to the temperature treatment in the DSC. In other samples the recrystallization peak was not observed, indicating the formation of β sheets after post-treatment with both EtOH and MeOH in all the other conditions. These results agree with those found in the FTIR ATR analysis.

Degradation peaks of the untreated films appear in the range of 270–272°C and for the treated films between 273–277°C, showing a slight increase in degradation temperatures in the treated samples. This can be attributed to the aforementioned increase in the percentage of crystalline structures in the treated samples, which is consistent with data reported in the literature (Um et al., 2001; Nogueira et al., 2010). These reports indicate a peak of degradation at lower temperatures in untreated samples compared to the degradation peaks of samples treated with solvents. This is due to the higher percentage of amorphous structures in the samples without treatment.

### Absorption Water and Weight Loss in Aqueous Medium

The weight loss and the water absorption in aqueous media of SFW films with and without post treatment were obtained. The results are shown in Table 5. Regarding weight loss, all samples show a greater mass loss on day 7 compared to day 1, as expected. However, the percentages are less than 10% in both cases. In addition, there is a statistically significant difference for days 1 and 7 in the weight loss of the treated samples compared to the untreated ones with a *p* value <0.05. The weight loss results on day 7 for the untreated samples (9.5%) were higher with regards to VEtOH 15 min (8.9%) followed by VEtOH 1 h (8.7%), IEtOH 15 min (8.4%), IEtOH 1 h (7.8%), VMeOH 15 min (7.4%), VMeOH 1 h (7.2%), IMeOH 1 min (6.2%), and finally IMeOH 1 h (5.3%). The values obtained for the untreated samples, and samples treated with IMeOH for 1 h are similar to those reported in the literature by Srivastava et al. (2015). Moreover, Mandal et al. (2009) and Zhou et al. (2016) reported a non-significant loss in IMeOH and 10% in untreated films. On the other hand, samples treated with EtOH showed a mass loss between 7 and 8%, a percentage comparable to that reported by Rajkhowa et al. (2011).

**TABLE 5:** Results of weight loss films and water absorption films of SFW.

	**Weight loss (%)**		**Water absorption (%)**	
	**D1**	**D7**	**D1**	**D7**
Untreated	8.52 ± 0.56	9.55 ± 0.32	30.65 ± 0.94	34.91 ± 0.39
VEtOH 15 min	7.70 ± 0.92	8.91 ± 0.11	29.32 ± 0.79	32.16 ± 0.51
VEtOH 1 h	7.15 ± 0.55	8.74 ± 0.20	27.66 ± 0.77	30.12 ± 0.04
IEtOH 15 min	6.47 ± 0.18	8.43 ± 0.19	25.47 ± 0.71	28.15 ± 0.57
IEtOH 1 h	5.88 ± 0.43	7.82 ± 0.53	22.26 ± 1.46	25,46 ± 0.26
VMeOH 15 min	4.47 ± 0.02	7.40 ± 0.22	19.23 ± 0.51	22.83 ± 0.34
VMeOH 1 h	4.13 ± 0.14	7.25 ± 0.28	16.82 ± 0.67	19.82 ± 0.95
IMeOH 15 min	2.82 ± 0.19	6.26 ± 0.29	14.14 ± 0.92	17.21 ± 0.64
IMeOH 1 h	1.64 ± 1.64	5.38 ± 5.38	12.70 ± 0.41	15.03 ± 0.05

The water absorption results show a similar trend to that which was previously observed, corresponding to degradation. There was a higher absorption of water observed in untreated samples (34.9%), followed by VEtOH 15 min (32.1%), VEtOH 1 h (30.1%), IEtOH 15 min (28.1%), IEtOH 1 h (25.4%), VMeOH 15 min (22.8%), VMeOH 1 h (19.8%), IMeOH 15 min (17.2%), and finally IMeOH 1 h (15%). This can be attributed to the fact that samples treated with MeOH have a higher percentage of crystalline structures, which makes them more hydrophobic compared to those treated with vapor and untreated samples. There is a statistically significant difference on day 7 in the treated samples compared to the untreated ones, with a *p* value <0.05. Srivastava et al. (2015), Bagrov et al. (2017), and Bie et al. (2015) reported absorption in samples treated with 15% MeOH and untreated between 36 and 46%, respectively. These values are similar to those obtained in this study.

### Scanning Electron Microscopy

**FIGURE 6:**
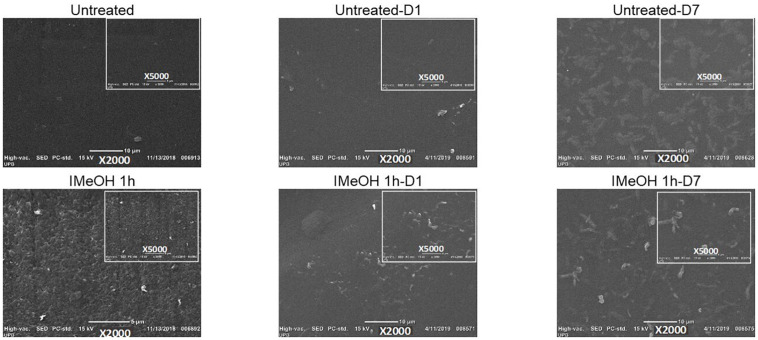
SEM untreated, treated films IMeOH 1 h, before and after the degradation.

Scanning electron microscopy micrographs (Figure 6) showed a change in the surface morphology of films treated by immersion in MeOH for 1 h, changing from a smooth to a rough surface, due to the contraction of the proteinaceous material. This phenomenon can be explained by the hydrophobic dehydration that occurs due to molecular interactions between protein chains and the polar solvent (Chen et al., 2009). These results are in agreement with those reported in the literature by Terada et al. (2016) and Wongpanit et al. (2007) who reported a rougher surface when using immersion post-treatments with more than 90% EtOH and MeOH respectively, which could potentially rebound in the cell adhesion (Wongpanit et al., 2007; Terada et al., 2016).

In addition, SFW films treated by immersion (Supplementary Figures 5, 6) show a higher roughness compared to those treated by saturated vapor (Supplementary Figures 7, 8), given the large density of molecules of solvent in the liquid state. This could suggest higher interaction between the SF film and the solvent. On the other hand, post-treatments with MeOH (Supplementary Figures 5, 7) cause a more noticeable change due to the higher polarity of MeOH compared to that of EtOH (Supplementary Figures 6, 8). This allows for easier interactions between water molecules and the solvent with higher polarity. The change in morphology is related to the structural and thermal changes discussed above. This is because the films that presented a greater apparent roughness in the SEM images are the same that presented a higher percentage of crystalline structures, along with a higher degradation temperature. Thus, this indicates that the immersion treatment with MeOH provides greater changes in the properties evaluated when compared to the other treatments.

The films morphology was also studied after the exposure of samples for 1 and 7 days, in the corresponding SEM images (Figure 6 and Supplementary Figures 5–8). It can be observed on day 1 what appears to be the swelling of the surface with a decrease in roughness, which can be attributed to the water absorption of the films. Moreover, on day 7, erosion is evident on the surface, showing some cavities and surface agglomerations caused by swelling and potentially by the solubilization process, which is consistent with results reported in the literature (Zhou et al., 2016; Wang et al., 2019). According to the content of secondary structures present in SFW films, there are significant changes in morphology (Minoura et al., 1990; Lu et al., 2010), with films post-treated with EtOH having the most evident differences. Those films show a higher content of amorphous structures, compared to those treated with MeOH, which favors higher weight loss values, as presented above.

**FIGURE 7:**
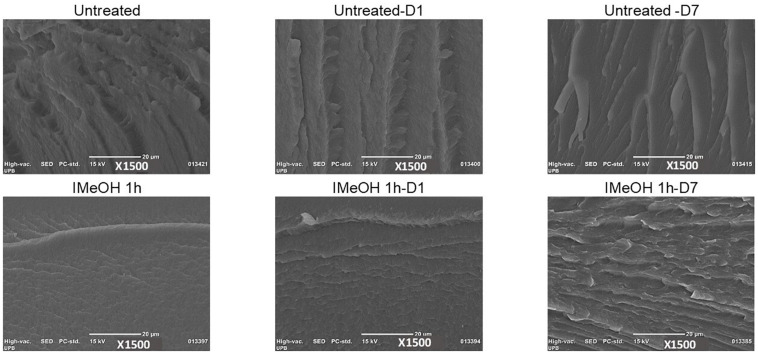
SEM cross-section untreated, treated films IMeOH 1 h, before and after the degradation.

The images obtained by SEM (Figure 7 and Supplementary Figures 9–12) of the cross-section show changes in the striations of the samples, with narrower filaments/striations and more compacting in the samples with a higher percentage of crystallinity (IMeOH, Supplementary Figure 9). In contrast, as the samples decrease in their percentage of crystallinity, the size of striae become wider (EtOH treated samples, Supplementary Figures 11, 12). These results confirm our observations regarding the dependence on the morphology of the films cross-sections and the percentage of the crystalline structures, and are also consistent with those previously reported (Zhang et al., 2012). Zhang et al. (2012) had shown that films with greater crystallinity present a more regular compact morphology.

## Conclusion

The effects of post-treatment with EtOH and MeOH at different times, by immersion and saturated atmosphere on the SFW films, were evaluated. The change in the percentage of secondary structures and their influence on degradation rates and thermal properties were determined. The results obtained indicate that the treatments evaluated in this study are effective in increasing the content of the crystalline structure, evidenced in the reduction of random coils and the increase of β sheets as demonstrated by XRD and FTIR. Therefore, treatments with organic solvents present an effective mechanism to regulate the crystallinity, water integrity and thermal stability of the material.

Among the post-treatments studied, immersion in MeOH for 1 h provided the highest increase in percentage of crystalline structures with a value of 57.1% compared to 33.3% of the sample without treatment. This increase also indicates that films with higher percentage of crystalline structures also show higher degradation temperatures, as well as decreased degradation and water absorption rates when compared to untreated samples.

These results are relevant for applications of SF from SFW in biomedicine, such as cellular scaffolding, as regulated degradation rates and mechanical and thermal stability are required to allow adhesion, cell proliferation, and durability of scaffolding.

Films obtained from fibrous wastes showed similar properties to those reported for SF extracted from cocoons, indicating that materials manufactured from wastes have the potential to be used in biomedical applications. Additionally, the results obtained from this study can aid in improving the sustainability of the sericultural chain, especially regarding small scale production, allowing for better utilization of resources and increased value of the material.

## Data Availability Statement

The datasets generated for this study are available on request to the corresponding author.

## Author Contributions

MP developed the materials and performed the experiments and characterizations. MP, MSP, and AR-O contributed to the conceptualization, data analysis, and writing of the manuscript. All authors listed have made a substantial, direct and intellectual contribution to the work, and approved it for publication.

## Conflict of Interest

The authors declare that the research was conducted in the absence of any commercial or financial relationships that could be construed as a potential conflict of interest.
