# Supplementary material for: Effects of Chemical Post-treatments on Structural and Physicochemical Properties of Silk Fibroin Films Obtained From Silk Fibrous Waste
Source: Front Bioeng Biotechnol. 2020 Dec 2;8:523949. doi: 10.3389/fbioe.2020.523949 (PMC7738614; doi:10.3389/fbioe.2020.523949)
Supplement: Supplementary file 1 [file Table_1.docx]

Supplementary Material





**Figure S1** – FTIR-ATR Spectra untreated and treated films with EtOH.





**Figure S2** - FTIR-ATR Spectra untreated and treated films with MeOH.


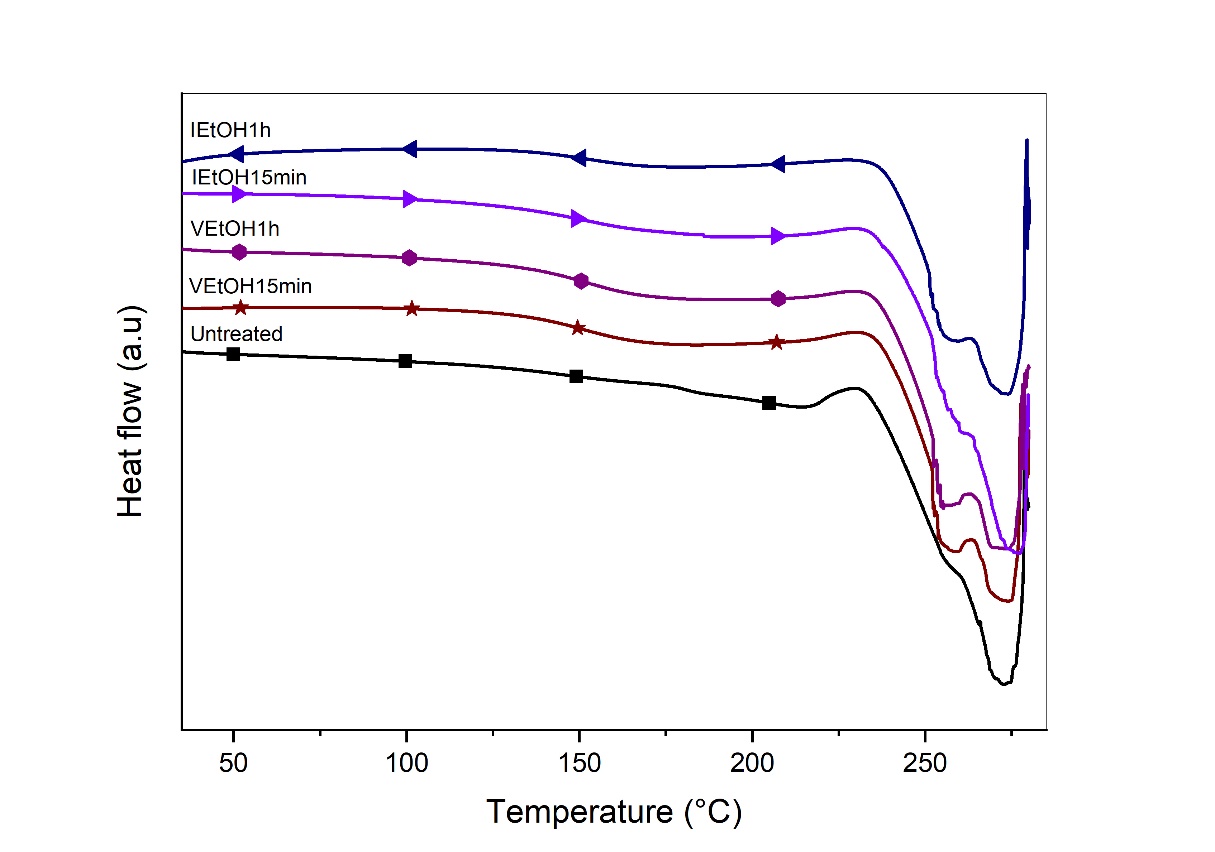


**Figure S3** - DSC Curves untreated and treated films with EtOH.


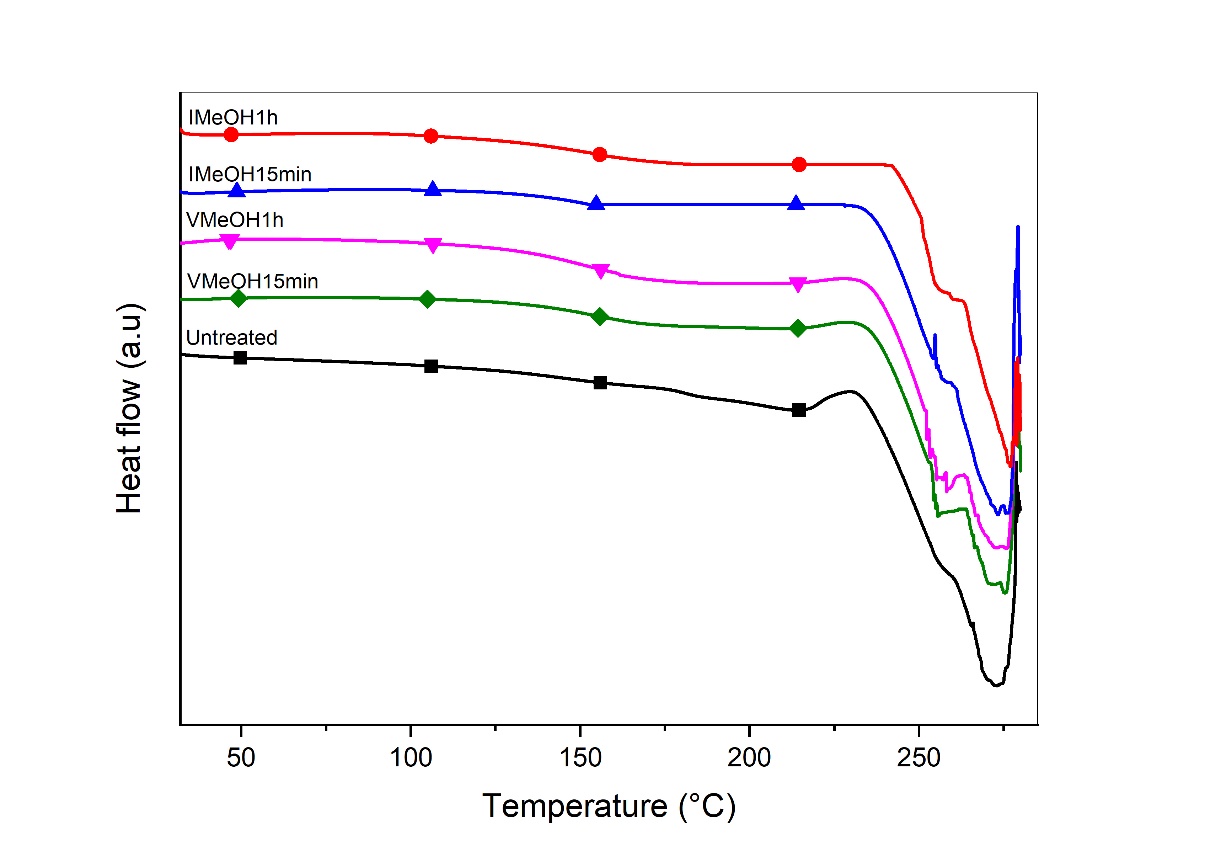


**Figure S4** - DSC Curves untreated and treated films with MeOH.


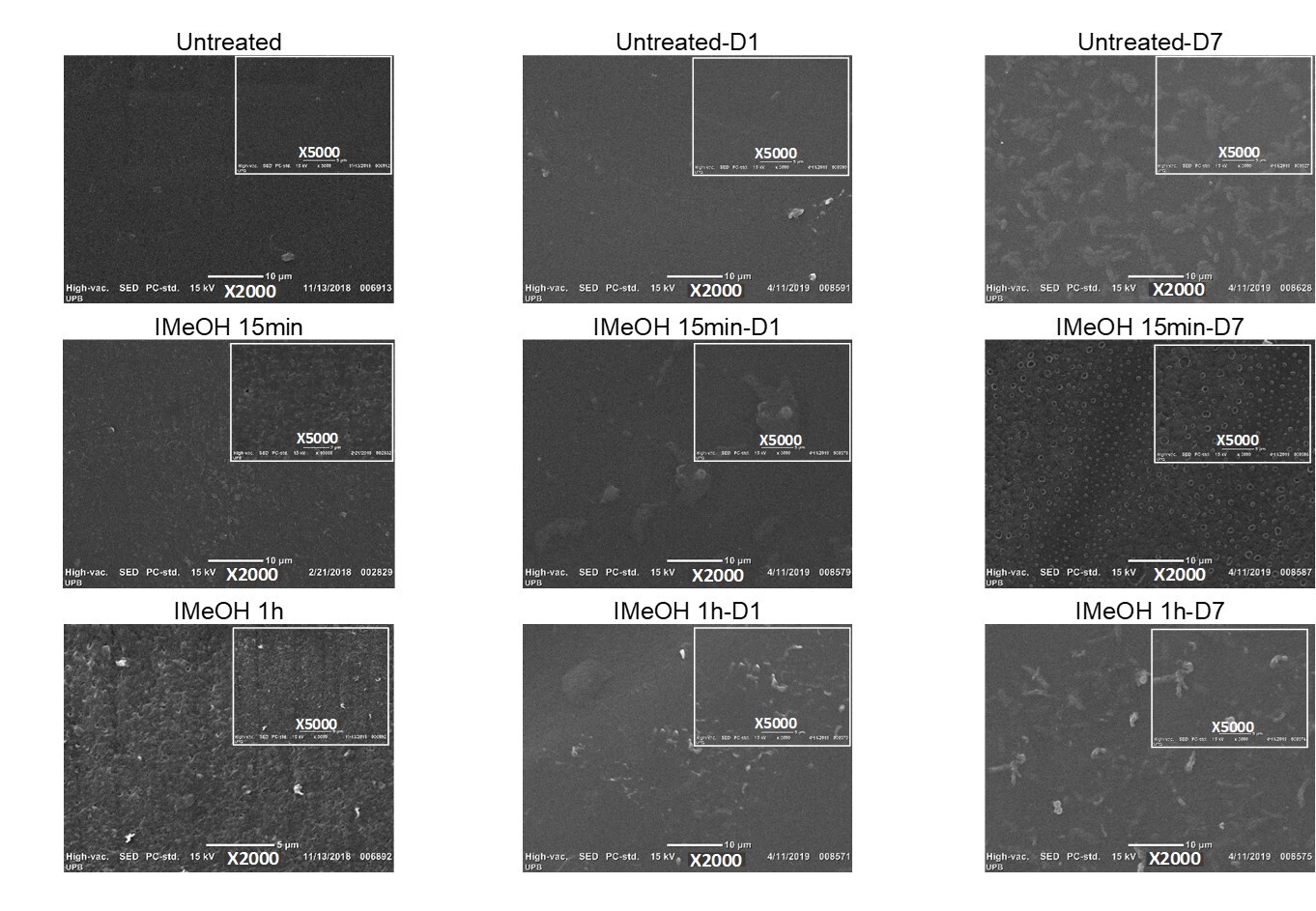


**Figure S5** – SEM untreated, treated films IMeOH, before and after the degradation.


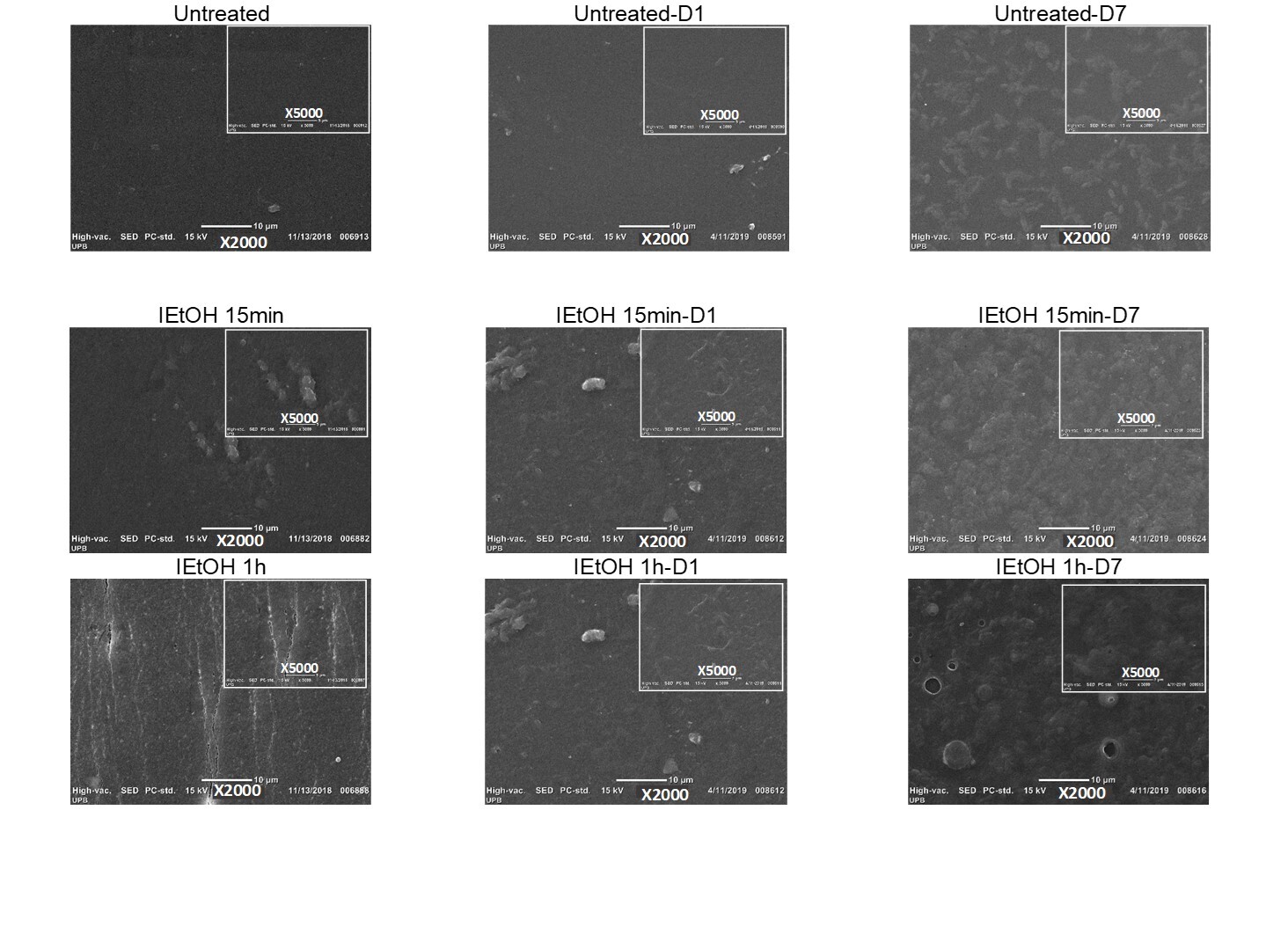


**Figure S6** – SEM untreated, treated films IEtOH, before and after the degradation.


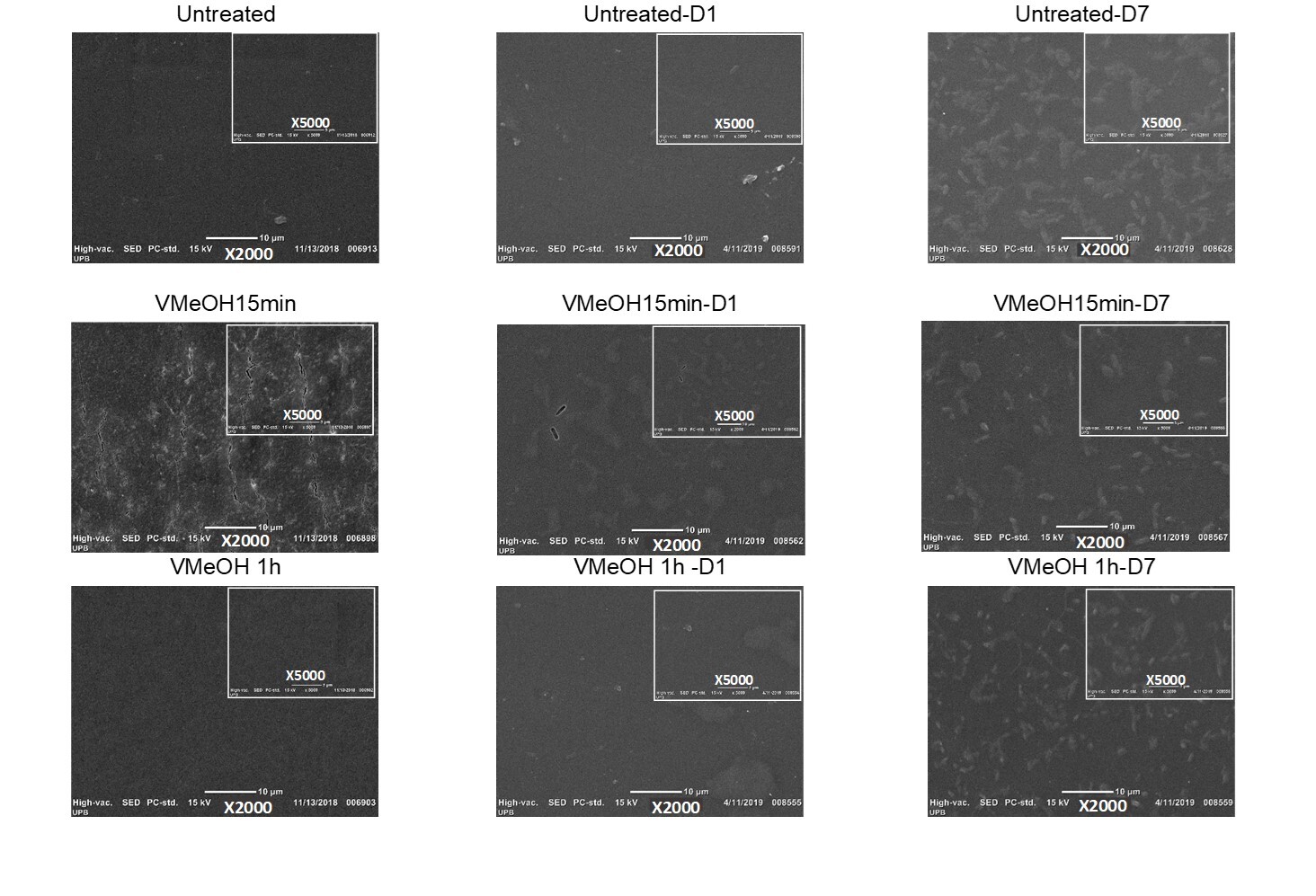


**Figure S7** – SEM untreated, treated films VMeOH, before and after the degradation.


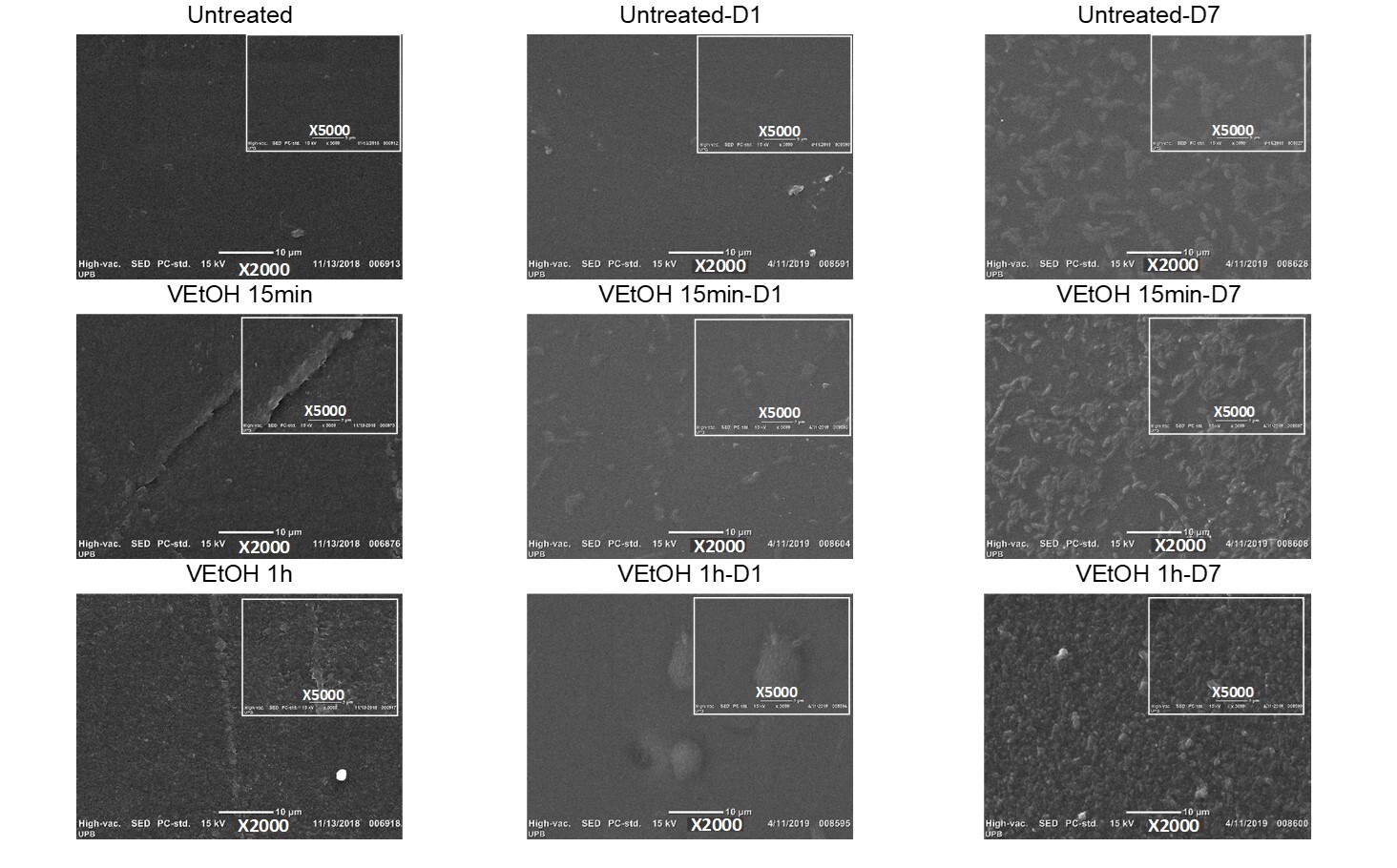


**Figure S8** – SEM untreated, treated films VEtOH, before and after the degradation.


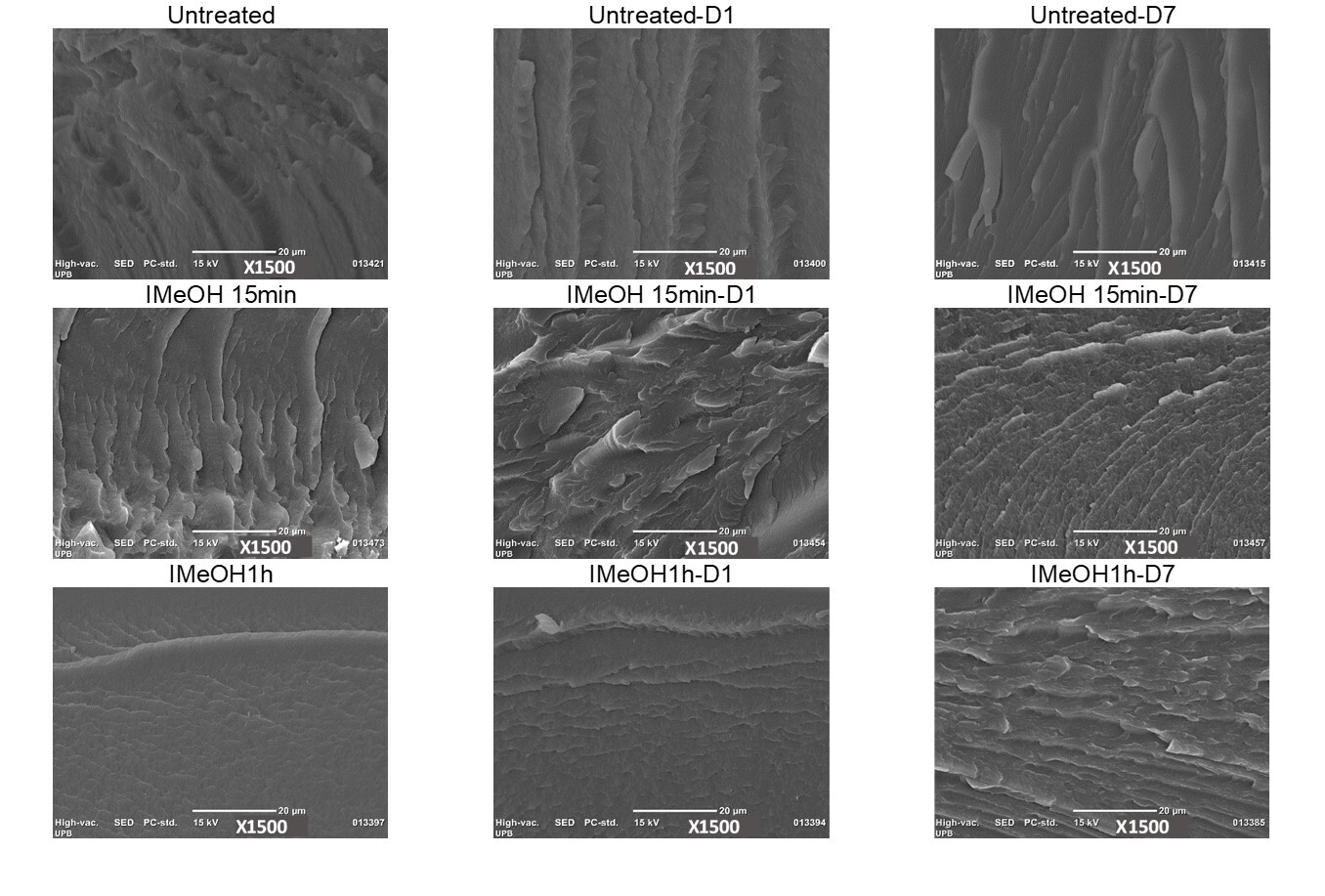


**Figure S9 –** SEM cross-section untreated, treated films IMeOH, before and after the degradation.


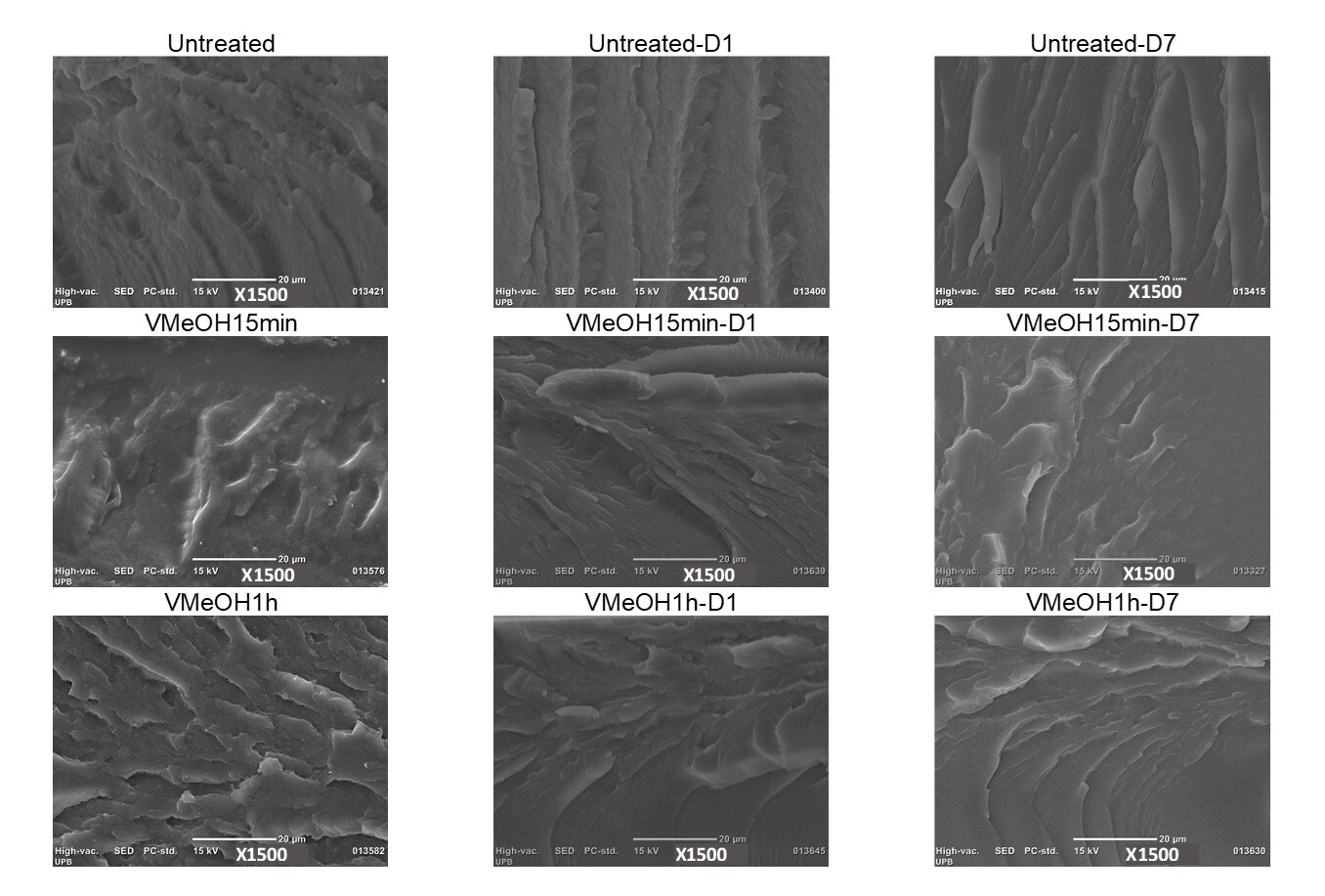


**Figure S10 –** SEM cross-section untreated, treated films VMeOH, before and after the degradation.


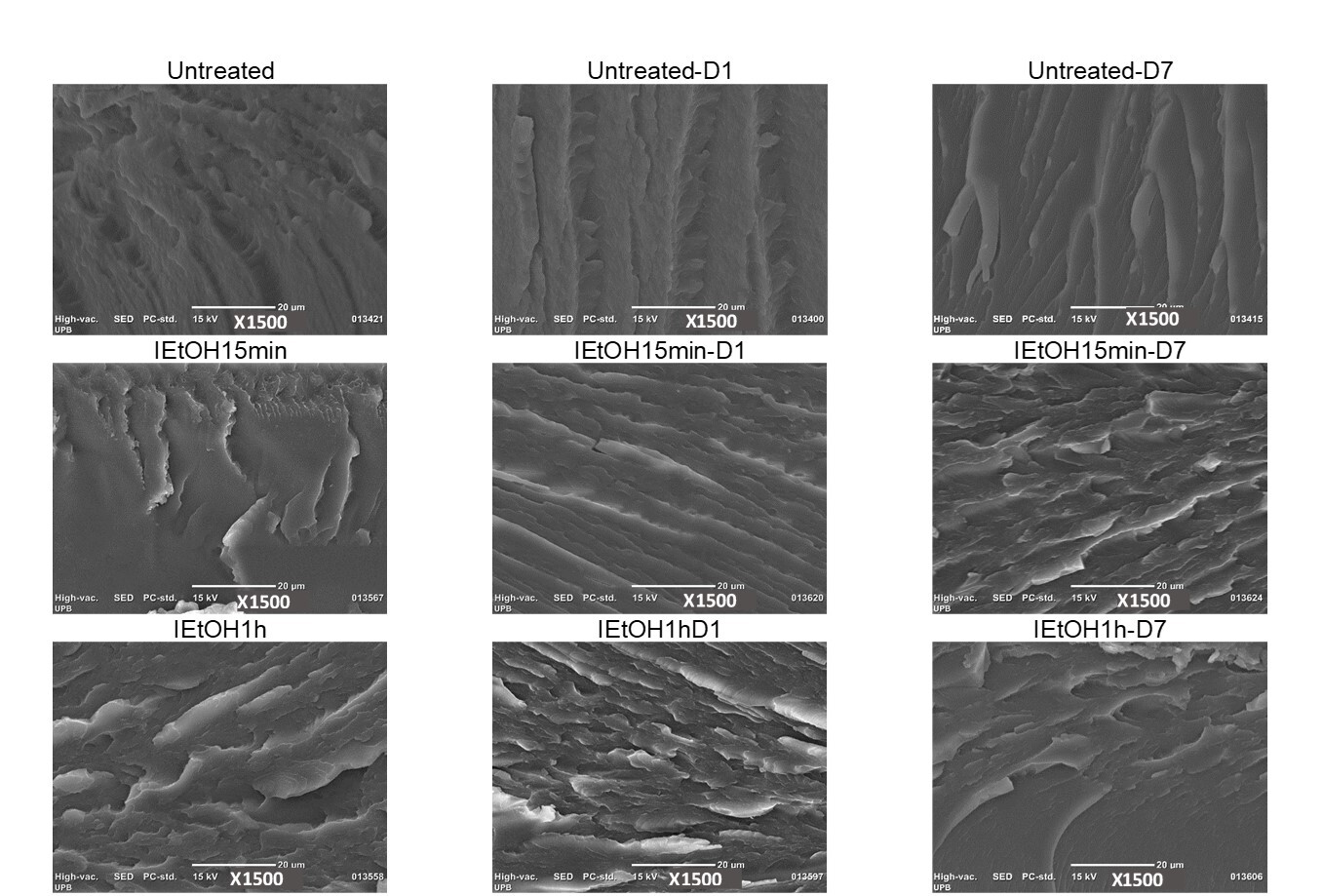


**Figure S11 –** SEM cross-section untreated, treated films IEtOH, before and after the degradation.


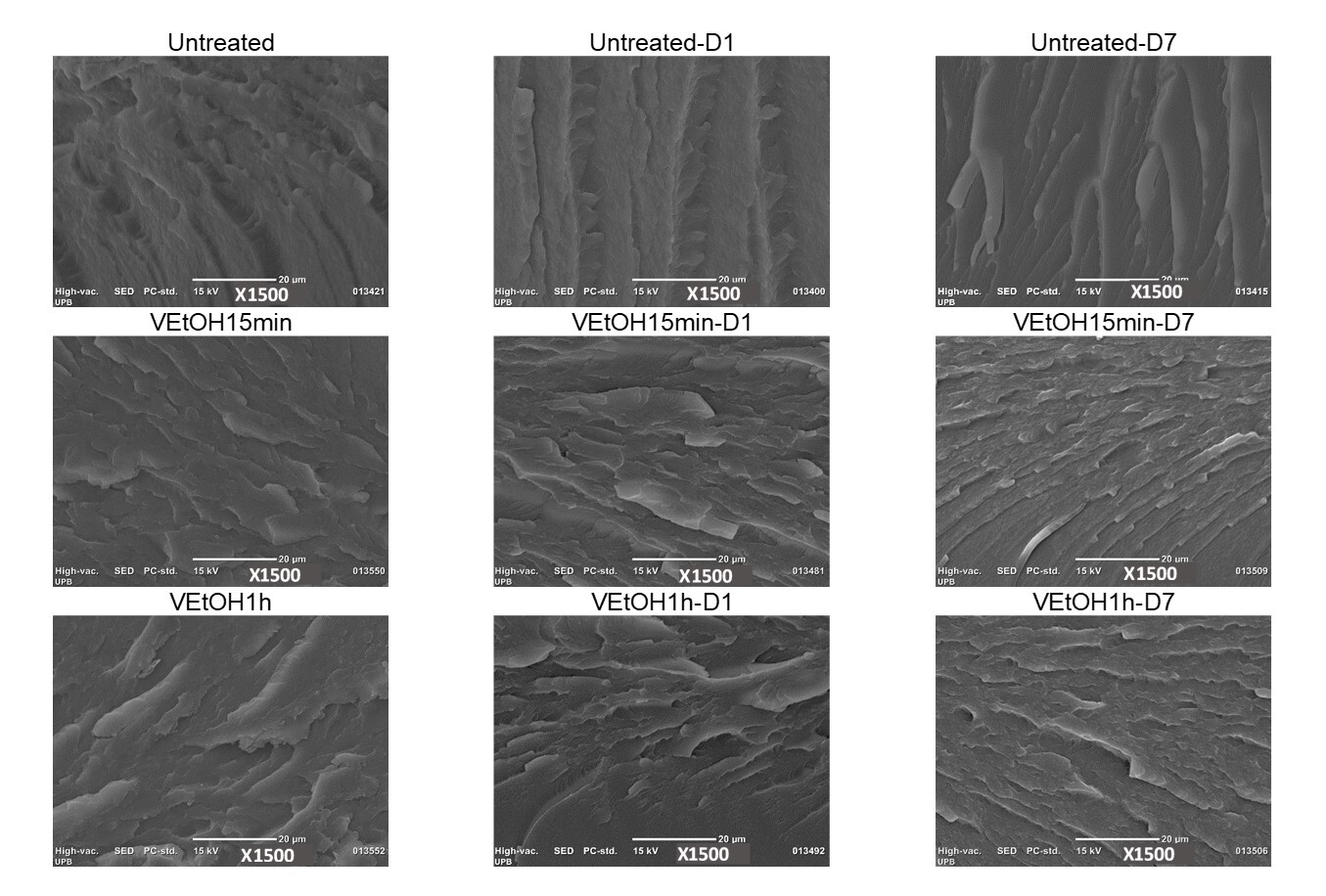


**Figure S12 –** SEM cross-section untreated, treated films VEtOH, before and after the degradation.
